# 17p deletion strongly influences rituximab elimination in chronic lymphocytic leukemia

**DOI:** 10.1186/s40425-019-0509-0

**Published:** 2019-01-29

**Authors:** Cristina Bagacean, Adrian Tempescul, David Ternant, Anne Banet, Nathalie Douet-Guilbert, Anne Bordron, Boutahar Bendaoud, Hussam Saad, Mihnea Zdrenghea, Christian Berthou, Gilles Paintaud, Yves Renaudineau

**Affiliations:** 1 U1227 B Lymphocytes and Autoimmunity, University of Brest; INSERM; networks IC-CGO and REpiCGO from “Canceropole Grand Ouest”, Brest, France; 2 Department of Hematology, Brest University Medical School Hospital, 5 Foch Avenue, BP 824, F-29609 Brest, France; 3 Laboratory of Immunology and Immunotherapy, Brest University Medical School Hospital, Brest, France; 4 University of Tours, EA 7501 Innovation and Cell Targeting Group, CHRU de Tours, Laboratory of Pharmacology-Toxicology, Tours, France; 5 Laboratory of Cytogenetics, Brest University Medical School Hospital, Brest, France; 6 “Iuliu Hatieganu” University of Medicine and Pharmacy, Cluj-Napoca, Romania

**Keywords:** Chronic lymphocytic leukemia, Anti-CD20 monoclonal antibody, Rituximab, Pharmacokinetics, Clearance, 17p deletion

## Abstract

Chronic lymphocytic leukemia (CLL) is the most common type of leukemia and the anti-CD20 monoclonal antibody, rituximab, represents the therapeutic gold standard for more than 2 decades in this pathology, when used in combination with chemotherapy. However, some patients experience treatment resistance or rapid relapses, and in particular, those harboring a 17p/TP53 deletion (del(17p)). This resistance could be explained by a chemo-resistance, but it could also result from the direct impact of del(17p) on the pharmacokinetics of rituximab, which represents the aim of the present study. Accordingly, 44 CLL patients were included in the study, and among them 9 presented a del(17p). Next, a total of 233 rituximab sera were selected for a pharmacokinetic study and analyzed in a two-compartment model showing important differences when del(17p) CLL patients were compared with non-del(17p) patients treated with rituximab and chemotherapy: (1) clearance of rituximab was faster; (2) central volume of rituximab distribution V1 (peripheral blood) was reduced while peripheral volume V2 (lymphoid organs and tissues) was increased; and (3) the rate of rituximab elimination (Kout) was faster. In contrast, the group with a better prognosis harboring isolated del(13q) presented a slower rate of elimination (Kout). Pharmacokinetic parameters were independent from the other factors tested such as age, sex, chemotherapy regimen (fludarabine/cyclophosphamide versus bendamustine), *IGHV* mutational status, and *FCGR3A* 158VF status. In conclusion, this study provides an additional argument to consider that del(17p) is effective not only to control chemoresistance but also monoclonal antibody activity, based on higher rituximab turnover.

## Introduction

In chronic lymphocytic leukemia (CLL), deletion (del) of the short arm of chromosome 17 (17p13) is found in 5 to 8% of patients requiring first-line treatment and is associated with rapid disease progression as well as a poor response to treatment with a median overall survival (OS) of 2 to 3 years from the time of first-line treatment [1–3]. Such an unfavorable prognosis has been attributed to the critical role played by TP53 on the apoptosis process control, and by halting the cell cycle to allow time for the DNA-repair system to catch up. Such an effect is reinforced by the observation that more than 80% of patients with CLL carrying del(17p) in one allele have a TP53 mutation on the remaining second allele, leading to complete loss of TP53 protein function. Moreover, 4 to 5% of CLL cases carry a TP53 mutation in the absence of del(17p), shortening the progression-free survival (PFS) and OS, similar to that seen in cases with del(17p) [4]. As a consequence, and in order to spare patients from ineffective treatment regimens, fluorescent in situ hybridization (FISH) for 17p13 and TP53 mutational analysis have been adopted into routine clinical diagnostics before treatment-initiation and at relapse.

Literature is also growing on the premise that TP53 affects the response to immunotherapy in a large panel of tumors [5–7]. The best studied monoclonal antibody (mAb) affected by the TP53 status is rituximab (RTX) an anti-CD20 mAb that improves patient outcome in B-cell malignancies, and it has been confirmed that CD20 is an important target in CLL [8]. Until recently, the gold standard in CLL treatment was based on the combination of RTX with chemotherapy (fludarabine and cyclophosphamide or bendamustine), but real progress has been made with the introduction of novel molecules such as key tumor tyrosine kinase inhibitors. Their association with anti-CD20 mAb and better knowledge on the therapeutic resistance mechanisms, including those related to TP53 loss, are works in progress with promising results.

RTX (MabThera®, Rituxan®) is a chimeric mAb, incorporating a human Kappa constant region, a human IgG1 Fc region plus a murine variable region, which recognizes the human CD20 antigen [9]. Several studies reported high variability among individuals in RTX pharmacokinetics which has been linked to several factors including tumor burden, *FCGR3A* polymorphism or a defective complement pathway [10–12]. The present study aimed to investigate the influence of TP53 loss on RTX pharmacokinetics in CLL patients.

### Study design

Clinical and biological data were available from 44 patients diagnosed with CLL according to the World Health Organization (WHO) classification between 1996 and 2011 at Brest University Hospital [13]. Consent was obtained from all individuals and the protocol approved by the Ethical Board (ClinicalTrials.gov: NCT03294980; CRB Brest, collection 2008–214), in accordance with the Declaration of Helsinki.

Serum concentrations of RTX were determined before each RTX infusion, as previously described [14]. A total of 233 sera were collected at the time of RTX infusion in patients receiving immunochemotherapy and analyzed using a two-compartment model with (i) non-specific (linear) and (ii) target-mediated (nonlinear) elimination pathways, as previously described [15].

## Results and discussion

### Population

A total of 44 CLL patients were included in the study. The median age at study entry was 72 years [36–85 years], 23 were male and 21 female and the three Binet stages were represented A (*n* = 5), B (*n* = 22), and C (*n* = 17). Twenty-eight patients were treated with a RTX, fludarabine, cyclophosphamide (RFC) regimen, and 17 with a RTX, bendamustine (RB) regimen as first-line therapies. The majority of the patients (40/44 [90.9%]) included in the study achieved a complete response (CR) and 4 patients, treated with RFC, presented a complete response with an incomplete bone marrow hematological recovery (CRi). None of these 4 patients presented a del(17p). The mean number of treatment courses administered was 5.3 [range 4–6] and 5.2 [4–6] for RFC and RB, respectively. After immuno-chemotherapy, the median follow-up was 55.5 months [24–108 months], median time to relapse (TTR) was 60 months [12 - more than 108 months], and time to second CLL treatment (TST) was 84 months [24 - more than 108 months]. In the group studied, 8 patients presented an isolated del(13q), 10 patients a trisomy 12, 4 a normal caryotype, 7 patients a del(11q) and 9 patients a del(17p). *FCGR3A*-158 V/F and the *IGHV* mutational status were available for all patients and 16 patients, respectively.

### Del(17p)/TP53 and RTX pharmacokinetics

**Fig. 1:**
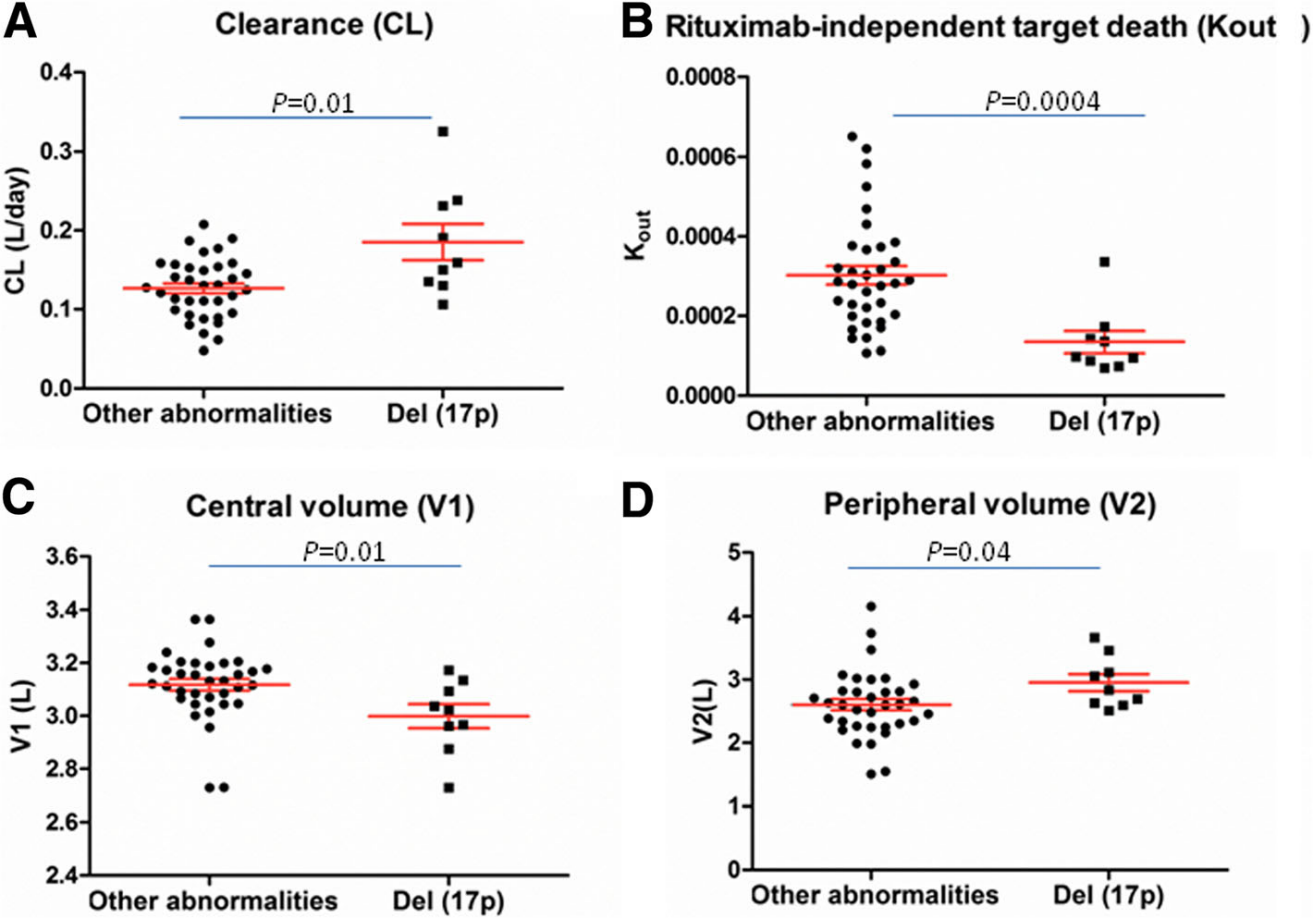
Del(17p) influences the pharmacokinetics of rituximab (RTX). Del(17p) CLL patients have a faster rituximab (RTX) clearance (CL) (**a**), a lower RTX distribution in the central volume (V1) (**c**) while peripheral volume (V2) was larger (**d**), and a significantly lower RTX rate of elimination (Kout) (**b**) compared to patients presenting other cytogenetic abnormalities. Statistics are indicated when *p* < 0.05 (Mann-Whitney U test)

TP53 loss represents an important negative predictor for response to immunotherapy not only in hematological diseases but also in solid tumors, thus supporting the concept that mAb pharmacokinetics may be affected by the TP53 status [3]. Accordingly, a well established 2-compartiment model was used showing important differences between CLL patients presenting or not a del(17p): (i) RTX clearance (CL) in del(17p) CLL patients was significantly higher than in non-del(17p) CLL patients (Fig. 1a, median CL = 0.16 L/day in del(17p) CLL versus 0.12 L/day in the CLL patients presenting other cytogenetic anomalies, *p* = 0.01). This higher CL is potentially caused by the impaired control of p53 on cell-cycle, apoptosis and RTX capacity to promote immune system recruitment/activation; (ii) V1 was reduced in del(17p) CLL patients which is in relation with the observation that peripheral del(17p) B-CLL cells present a loss of CD20 [16, 17] (Fig. 1c, *p* = 0.01); (iii) V2 was increased, supporting a more extensive tumor antigen burden available for RTX in the lymphoid organs (Fig. 1d, *p* = 0.04). This is in relation to the presence of expanded proliferation centers in lymph nodes of patients with TP53 loss [18]; and (iv) Kout which is lower in del(17p) CLL patients (*p* = 0.0004) suggesting a more rapid RTX elimination in these patients (Fig. 1b).

### Other parameters

In order to complete the pharmacokinetic analysis we next integrated the data concerning age, sex, body surface area (BSA), the traditional staging system Binet, treatment protocol, tumor burden (lymphocytosis, lymphoadenopathy areas), cytogenetic aberrations, mutational status of the *IGHV*, and *FCGR3A* status (Table 1). After adjustment for multiple testing using the Benjamini–Hochberg method, significant differences concern Kout, that was decreased in CLL patients harboring a del(17p) (*p* = 0.007). Interestingly, for CLL patients with isolated del(13q), and referred as patients with good therapeutic response, the Kout was significantly increased (*p* = 0.02). Both del(17p) and del(13q) control apoptosis, but differences are related to the cell cycle control, and the capacity to activate the immune system which are both affected by the loss of TP53. Moreover, in non-del(17p) CLL patients, the absence of an association between *FCGR3A* genotypes and pharmacokinetic parameters further reinforces complement instead of Fc gamma dependent mechanisms for RTX elimination in CLL. This assertion is supported by a recent report showing the potential implication of TP53 loss in complement activation control [12, 19, 20].

**Table 1:** Univariate analysis of pharmacokinetic parameters and biological or clinical variables

		CL(L/day)	Kout (day-1)	V1(L)	V2(L)
	*n*	*p*			
Age	44	0.86	0.82	0.90	0.99
Sex male versus female	23 versus 21	0.59	0.53	0.34	0.37
Body surface area (BSA, m^2^)	44	0.59	0.82	0.76	0.99
Binet stage A or B versus C	27 versus 17	0.89	0.82	0.34	0.99
Treatment RB versus RFC	17 versus 28	0.48	0.82	0.76	0.99
Lymphocytosis	44	0.59	0.30	0.96	0.99
Areas of lymphadenopathy	44	0.59	0.39	0.96	0.51
CD38 positivity (≥30%) versus negativity	21 versus 22	0.88	0.82	0.90	0.99
FCGR F/F versus V/F and *V*/V	25 versus 19	0.86	0.68	0.90	0.99
IGHV unmutated versus mutated status	10 versus 6	0.37	0.82	0.96	0.99
Cytogenetics					
Isolated del(13q) versus others	8 versus 36	0.37	0.02	0.76	0.23
Trisomy 12 versus others	10 versus 34	0.59	0.92	0.76	0.37
Normal caryotype versus others	4 versus 40	0.89	0.39	0.96	0.66
Del(11q) versus others	10 versus 34	0.37	0.17	0.76	0.17
Del(17p) versus others	9 versus 35	0.17	0.007	0.17	0.23
Time to relapse (TTR)	44	0.59	0.82	0.96	0.99
Time to second treatment (TST)	44	0.37	0.39	0.96	0.99

*vs*. versus, *CL* clearance, *Kout* first-order rate constant of rituximab independent death of latent target antigen, *V1* central distribution volume, *V2* peripheral distribution volume, *RB* rituximab bendamustine, *RFC* rituximab fludarabine cyclophosphamide, *FCGR* Fc gamma receptor, *IGHV* immunoglobulin heavy chain variable region. Values were adjusted for multiple testing using the Benjamini–Hochberg method (https://www.sdmproject.com/utilities/?show=FDR), and *p* < 0.05 considered as significant

## Conclusions

Our study supports the hypothesis that del(17p)/TP53 is not only important in protecting tumor cells from DNA damaging agents such as fludarabine and bendamustine but is also important for controlling RTX pharmacokinetics. Accordingly, this study provides an explanation for the RTX resistance observed in CLL patients presenting a del(17p) [3]. Further studies are now needed to test whether this effect is restricted to RTX in order to propose a more efficient anti-CD20 mAb in association with specific B cell inhibitors at treatment initiation in patients with del(17p) or TP53 mutations. Treatment of CLL patients with a deficient TP53 requires compounds that promote cell death independently of TP53. Two mAbs have this potential: obinituzimab, a glycocoengineered type-II anti-CD20 mAb, and alemtuzumab, an anti-CD52 which is no longer licensed for treatment of CLL. Another option is to reverse the capacity of the TP53 deficient tumor cells to control the immune system as highlighted by the success of the anti-PDL1 mAb in neoplastic B-cells from Richter syndrome (80% TP53 deletion/mutation). In Richter syndrome, TP53 loss induces PD-1 expression, explaining the higher expression of PD-1 in these patients and the higher efficacy of the anti-PDL1 mAb [21, 22].
